# Supplementary material for: Differential gene expression in skeletal organic matrix proteins of scleractinian corals associated with mixed aragonite/calcite skeletons under low mMg/Ca conditions
Source: PeerJ. 2019 Jul 15;7:e7241. doi: 10.7717/peerj.7241 (PMC6637933; doi:10.7717/peerj.7241)
Supplement: Supplemental Information 1 — Lists of up-regulated and down-regulated genes. [file peerj-07-7241-s001.pdf]

Table S1 Top 50 most significantly up-regulated (a) and down-regulated (b) genes, showing expression pattern for each gene between control and low-Mg conditions (deepening intensity of color indicating higher expression value). Estimated m-values (log 2(low-Mg/control)), and p-values for detecting differentially expressed genes from FPKM and annotation information (BLASTx results against NCBI non redundant database and the e-value) also shown (e-value cutoffs < 1e-6).

(a) Top 50 most up-regulated genes in low-Mg/Ca condition

contig name	blastx (Uniplot-Splot) annotation results	blastx e-value	Ca/Mg=5.2_rep1	Ca/Mg=5.2_rep2	Ca/Mg=0.5_rep1	Ca/Mg=0.5_rep2	m.value	p.value
TRINITY_DN199157_c0_g1_i1	Galaxin	6.00E−08	17.26	25.46	1494.27	1685.51	6.22	1.28E−139
TRINITY_DN215379_c0_g1_i1	Cytolysin−3	5.00E−08	165.49	153.96	1447.53	1716.73	3.31	3.96E−67
TRINITY_DN199692_c0_g1_i1	Uncharacterized skeletal organic matrix protein 5	3.00E−45	2163.93	2387.71	9071.10	11361.98	2.17	3.99E−42
TRINITY_DN183007_c0_g1_i2	Golgi−associated plant pathogenesis−related protein 1	2.00E−25	448.44	522.19	2223.13	2145.31	2.17	6.13E−42
TRINITY_DN180313_c0_g1_i1	Gremlin−1	1.00E−08	19.95	20.02	596.02	381.48	4.61	5.26E−37
TRINITY_DN189428_c0_g1_i1	Solute carrier family 23 member 2	4.00E−178	14183.87	17310.48	57189.57	64842.10	1.95	7.85E−37
TRINITY_DN196884_c0_g5_i1	Integrin alpha−2 (Fragment)	3.00E−07	1507.47	1476.77	5432.67	7513.27	2.12	1.11E−33
TRINITY_DN196327_c4_g1_i1	72 kDa type IV collagenase	4.00E−07	2063.87	2170.84	6511.77	6765.39	1.65	1.67E−32
TRINITY_DN189366_c2_g1_i1	Protein DD3−3	5.00E−67	13661.09	16845.45	51253.66	62436.10	1.90	1.91E−31
TRINITY_DN187629_c0_g1_i1	CUB and peptidase domain−containing protein 1 (Fragment)	4.00E−63	710.40	559.79	2252.01	2510.12	1.91	1.56E−29
TRINITY_DN184026_c0_g1_i2	Sushi, von Willebrand factor type A, EGF and pentraxin domain−containing protein 1	5.00E−29	204.54	271.89	1024.66	1303.90	2.29	6.44E−28
TRINITY_DN170829_c0_g1_i1	Serine protease 23	5.00E−40	1293.24	1235.96	3646.86	4657.67	1.72	3.18E−26
TRINITY_DN202817_c2_g2_i4	L−tyrosine decarboxylase	4.00E−13	2375.34	2662.00	6880.96	7796.53	1.54	5.30E−26
TRINITY_DN200370_c1_g1_i1	Protocadherin Fat 4	2.00E−21	588.41	1003.39	4483.34	3574.53	2.34	1.73E−24
TRINITY_DN200396_c1_g8_i1	Protein DD3−3	2.00E−66	334.79	286.25	1115.72	1070.91	1.82	2.56E−24
TRINITY_DN195315_c3_g1_i1	Skeletal aspartic acid−rich protein 2 (Fragment)	6.00E−160	334.95	359.01	1201.31	1071.72	1.71	1.30E−22
TRINITY_DN194222_c0_g3_i1	Spondin−1	1.00E−26	1740.90	1498.58	4646.84	4131.95	1.44	1.88E−21
TRINITY_DN196323_c0_g1_i1	Carbonic anhydrase 2	3.00E−52	7232.64	7765.78	17917.72	17866.41	1.25	1.77E−20
TRINITY_DN188586_c0_g1_i1	Retinal homeobox protein Rx2	2.00E−42	809.07	702.72	1962.56	2204.17	1.46	2.71E−20
TRINITY_DN201678_c0_g1_i1	Toxin PsTX−60B	2.00E−51	79.53	67.46	378.74	431.35	2.46	1.34E−18
TRINITY_DN203301_c4_g1_i3	Centrosomal protein of 192 kDa	1.00E−98	5225.24	6343.04	14403.23	13288.04	1.26	3.02E−17
TRINITY_DN202461_c5_g1_i1	Neuronal pentraxin−2	3.00E−13	92.19	108.65	468.49	428.86	2.16	4.12E−17
TRINITY_DN187529_c0_g1_i1	Homeobox protein HMX3−B	2.00E−34	1441.69	1269.12	3150.95	3234.43	1.24	4.19E−17
TRINITY_DN37452_c0_g1_i1	Homeobox protein DLX−6	1.00E−21	761.83	741.45	1712.72	1916.62	1.27	1.52E−16
TRINITY_DN198633_c1_g2_i2	Cholesterol oxidase	9.00E−10	7607.47	6013.61	16625.40	15743.52	1.25	3.98E−16
TRINITY_DN197325_c4_g1_i1	Signal transducing adapter molecule 2	5.00E−07	437.60	485.69	1120.45	1378.36	1.44	6.69E−16
TRINITY_DN197462_c0_g2_i1	Trace amine−associated receptor 7a	9.00E−13	106.75	98.52	414.08	439.20	2.06	8.43E−16
TRINITY_DN203041_c0_g3_i1	Collagen alpha−6(VI) chain	2.00E−07	1242.86	1028.78	2618.51	2737.08	1.24	1.36E−15
TRINITY_DN189073_c1_g1_i1	Uncharacterized skeletal organic matrix protein 8	1.00E−10	435.36	437.39	1046.35	1282.82	1.42	1.41E−15
TRINITY_DN202268_c1_g1_i1	Ankyrin−3	7.00E−09	345.49	377.31	905.20	1176.76	1.53	1.51E−15
TRINITY_DN201090_c2_g1_i1	Latrophilin−2	4.00E−32	1648.06	2034.55	4218.71	4360.37	1.22	1.57E−15
TRINITY_DN188453_c1_g2_i1	Ectin (Fragment)	7.00E−26	486.81	383.07	1115.91	1474.15	1.57	2.23E−15
TRINITY_DN190857_c1_g1_i1	Uncharacterized skeletal organic matrix protein 5	5.00E−53	1943.75	1648.82	3905.30	4818.87	1.28	2.30E−15
TRINITY_DN201678_c0_g3_i2	Toxin AvTX−60A	1.00E−32	75.63	101.67	384.85	422.17	2.19	3.54E−15
TRINITY_DN201210_c2_g6_i1	Uncharacterized skeletal organic matrix protein 5	2.00E−93	39.76	29.55	221.14	320.65	2.97	4.70E−15
TRINITY_DN204034_c0_g1_i2	Multiple epidermal growth factor−like domains protein 6	4.00E−38	1573.86	1550.02	3318.21	3358.61	1.10	4.82E−15
TRINITY_DN202096_c3_g1_i2	Oncoprotein−induced transcript 3 protein	5.00E−19	612.09	651.14	1536.73	1424.15	1.23	7.58E−15
TRINITY_DN203274_c1_g1_i1	Chondroitin sulfate ABC exolyase	3.00E−49	848.89	1140.26	2412.90	2598.59	1.33	7.80E−15
TRINITY_DN178459_c1_g1_i2	Hemicentin−1	4.00E−75	0.00	0.00	240.03	54.44	8.05	1.32E−14
TRINITY_DN199057_c0_g1_i2	Anoctamin−3	3.00E−164	168.02	243.54	648.12	753.12	1.77	1.37E−14
TRINITY_DN172840_c0_g2_i1	Peroxidase mlt−7	1.00E−08	12365.23	10569.30	24574.98	34641.27	1.37	1.47E−14
TRINITY_DN196807_c9_g5_i7	Melanotransferrin	5.00E−39	1036.60	1014.61	2138.80	2376.22	1.14	1.86E−14
TRINITY_DN197150_c2_g1_i1	Protein Wnt−8b	2.00E−63	173.88	238.40	709.39	627.95	1.70	2.61E−14
TRINITY_DN180711_c0_g1_i1	Oncoprotein−induced transcript 3 protein	2.00E−14	494.58	403.66	1113.47	1152.12	1.33	3.03E−14
TRINITY_DN36562_c0_g1_i1	Peroxidase−like protein 3 (Fragment)	6.00E−22	554.24	430.33	1285.29	1227.17	1.35	3.13E−14
TRINITY_DN196454_c2_g2_i3	Macrophage mannose receptor 1	1.00E−07	129.87	125.67	432.17	469.34	1.82	5.62E−14
TRINITY_DN196807_c9_g5_i2	Serotransferrin (Fragment)	1.00E−35	4745.68	5188.12	9761.70	12352.55	1.15	1.03E−13
TRINITY_DN184768_c1_g1_i2	Meprin A subunit beta	3.00E−44	840.73	727.46	1738.53	1728.91	1.14	2.16E−13
TRINITY_DN190741_c0_g1_i1	Bifunctional arginine demethylase and lysyl−hydroxylase JMJD6	6.00E−07	704.20	674.64	1456.80	1605.78	1.15	2.23E−13
TRINITY_DN198008_c0_g1_i1	Amiloride−sensitive sodium channel subunit alpha	3.00E−45	1956.77	1765.30	3712.65	3994.89	1.05	2.73E−13

(b) Top 50 most down-regulated genes in low-Mg/Ca condition

contig name	blastx (Uniplot-Splot) annotation results	blastx e-value	Ca/Mg=5.2_rep1	Ca/Mg=5.2_rep2	Ca/Mg=0.5_rep1	Ca/Mg=0.5_rep2	m.value	p.value
TRINITY_DN193151_c3_g6_i1	Dermatopontin	1.00E-20	2601.13	1833.80	156.02	156.68	-3.83	3.81E-71
TRINITY_DN191891_c0_g1_i1	Cytochrome P450 1A1	5.00E-85	1914.21	2059.64	401.98	302.81	-2.50	1.03E-42
TRINITY_DN196708_c0_g1_i3	Hemicentin-2	3.00E-20	1484.30	991.42	159.17	101.27	-3.25	3.30E-35
TRINITY_DN193616_c5_g6_i1	GFP-like fluorescent chromoprotein amFP486	2.00E-108	1850.53	1855.93	552.97	522.59	-1.78	7.16E-31
TRINITY_DN199796_c2_g3_i1	Tenascin-R	4.00E-59	2527.72	1850.80	419.42	522.35	-2.22	4.70E-30
TRINITY_DN193151_c1_g1_i2	Millepora cytotoxin-1	2.00E-11	205.88	165.27	0.00	0.00	-8.48	2.26E-29
TRINITY_DN182245_c0_g1_i1	D-galactoside-specific lectin	3.00E-17	3591.38	2408.68	625.28	672.33	-2.21	4.36E-29
TRINITY_DN199796_c2_g3_i6	Tenascin-R	3.00E-23	1803.04	1740.67	422.91	544.12	-1.87	2.41E-27
TRINITY_DN193151_c1_g1_i1	Millepora cytotoxin-1	4.00E-10	271.85	488.16	19.57	12.91	-4.55	1.15E-26
TRINITY_DN199796_c2_g1_i3	Fibrinogen C domain-containing protein 1	2.00E-30	3080.48	2321.74	579.78	764.36	-2.01	1.96E-25
TRINITY_DN193616_c5_g6_i3	GFP-like fluorescent chromoprotein amFP486	1.00E-87	4135.23	3680.43	1407.16	1430.95	-1.46	8.85E-24
TRINITY_DN189449_c2_g9_i1	Collagen triple helix repeat-containing protein 1	4.00E-39	2460.16	1414.94	368.15	371.68	-2.39	1.48E-23
TRINITY_DN202253_c1_g8_i6	Short-chain collagen C4 (Fragment)	3.00E-08	1783.09	1639.90	481.63	597.55	-1.67	1.05E-22
TRINITY_DN185833_c2_g1_i3	Olfactomedin-like protein 2A	4.00E-50	2291.16	1860.93	595.54	704.36	-1.68	1.66E-22
TRINITY_DN189418_c0_g1_i1	Tumor necrosis factor	2.00E-08	886.50	706.47	159.14	208.74	-2.11	1.98E-21
TRINITY_DN189511_c2_g1_i1	Integrin alpha-M	1.00E-11	469.32	466.68	44.01	85.90	-2.85	1.40E-19
TRINITY_DN194113_c0_g3_i1	Histone H4	3.00E-11	8864.46	6166.39	2029.53	2532.36	-1.72	2.39E-19
TRINITY_DN200741_c0_g3_i1	Tumor protein 63	4.00E-39	1745.93	942.83	263.57	268.12	-2.34	3.49E-19
TRINITY_DN202274_c7_g4_i4	Uncharacterized skeletal organic matrix protein 5	1.00E-11	1680.41	1382.80	398.47	546.54	-1.70	2.08E-18
TRINITY_DN185833_c2_g1_i1	Olfactomedin-like protein 2A	1.00E-51	7582.95	4773.85	1643.80	1906.57	-1.80	2.42E-18
TRINITY_DN202274_c7_g5_i1	Uncharacterized skeletal organic matrix protein 5	6.00E-14	1113.34	754.95	214.46	274.47	-1.93	6.57E-17
TRINITY_DN203609_c2_g1_i10	Leucine-rich repeat-containing protein 15	6.00E-31	407.82	557.73	108.16	92.73	-2.26	6.87E-17
TRINITY_DN202274_c7_g4_i3	Uncharacterized skeletal organic matrix protein 5	5.00E-31	1472.83	917.38	241.29	332.91	-2.06	8.30E-17
TRINITY_DN196686_c2_g1_i2	ETS domain-containing protein Elk-1	9.00E-24	1840.85	1368.72	503.26	586.17	-1.56	9.71E-17
TRINITY_DN198919_c2_g4_i4	Soma ferritin	7.00E-40	6097.09	6521.73	2482.96	2969.96	-1.21	3.09E-16
TRINITY_DN197036_c0_g1_i1	E3 ubiquitin-protein ligase TRIM71	8.00E-82	971.92	518.44	160.00	120.49	-2.41	1.58E-15
TRINITY_DN196910_c2_g2_i5	Tenascin-R	3.00E-56	275.70	273.68	45.68	39.53	-2.69	1.77E-15
TRINITY_DN199796_c2_g1_i4	Angiopoietin-related protein 4	2.00E-20	2144.39	1567.25	364.07	618.59	-1.92	1.79E-15
TRINITY_DN198375_c4_g1_i1	Tetratricopeptide repeat protein 28	1.00E-09	327.74	219.84	35.90	19.93	-3.29	1.83E-15
TRINITY_DN196074_c9_g4_i1	Tetratricopeptide repeat protein 28	2.00E-19	329.93	288.15	59.03	46.97	-2.54	2.60E-15
TRINITY_DN202721_c0_g4_i3	Cytochrome P450 1A1	2.00E-41	322.25	326.08	56.79	68.13	-2.38	3.44E-15
TRINITY_DN203658_c4_g9_i1	Fibroblast growth factor receptor	8.00E-61	4513.72	4860.64	2266.57	2054.69	-1.12	4.33E-15
TRINITY_DN192648_c1_g1_i1	Retinol dehydrogenase 8	6.00E-70	1705.74	1345.61	572.55	612.65	-1.36	4.55E-15
TRINITY_DN197899_c2_g2_i5	Olfactomedin-like protein 2B	2.00E-43	1951.03	1179.49	498.89	388.16	-1.82	1.54E-14
TRINITY_DN197899_c2_g2_i4	Olfactomedin-like protein 2B	2.00E-44	8535.95	4447.78	1985.56	1754.41	-1.80	3.88E-13
TRINITY_DN196910_c2_g2_i1	Tenascin-R	2.00E-71	539.02	580.01	149.78	200.26	-1.68	4.36E-13
TRINITY_DN203658_c4_g3_i1	Macrophage colony-stimulating factor 1 receptor	6.00E-19	7212.27	7350.54	3775.41	2963.37	-1.11	5.25E-13
TRINITY_DN189449_c2_g9_i3	Collagen triple helix repeat-containing protein 1	6.00E-34	807.74	844.77	356.51	295.78	-1.34	6.95E-13
TRINITY_DN200043_c2_g1_i1	E3 ubiquitin-protein ligase MIB2	9.00E-09	592.49	400.25	52.00	121.72	-2.51	5.66E-12
TRINITY_DN201951_c0_g1_i1	Glycine betaine transporter OpuD	1.00E-92	2463.91	2919.96	1206.84	1359.76	-1.07	7.78E-12
TRINITY_DN196332_c2_g1_i7	CD209 antigen-like protein D	9.00E-09	722.64	462.71	106.18	179.40	-2.05	1.08E-11
TRINITY_DN179002_c0_g3_i1	Proto-oncogene tyrosine-protein kinase receptor Ret	5.00E-33	757.10	575.93	252.16	234.40	-1.45	1.39E-11
TRINITY_DN194754_c1_g1_i2	Heat shock factor protein 1	1.00E-63	3562.00	3174.10	1632.89	1782.01	-0.98	2.21E-11
TRINITY_DN202274_c7_g5_i2	Uncharacterized skeletal organic matrix protein 5	9.00E-15	1582.55	1212.59	424.32	630.26	-1.41	4.17E-11
TRINITY_DN188280_c2_g2_i1	Collagen triple helix repeat-containing protein 1	3.00E-47	687.59	595.44	215.83	271.11	-1.40	4.41E-11
TRINITY_DN203204_c1_g2_i1	Tetratricopeptide repeat protein 28	8.00E-08	496.74	381.56	133.64	139.94	-1.68	4.54E-11
TRINITY_DN184423_c0_g1_i1	Mitochondrial import receptor subunit TOM22 homolog	8.00E-12	2222.11	1855.47	863.70	1051.38	-1.09	8.00E-11
TRINITY_DN184230_c2_g1_i1	Hemicentin-1	3.00E-23	124.26	72.51	5.81	1.00	-4.85	1.37E-10
TRINITY_DN164643_c1_g1_i1	Insoluble matrix shell protein 1 (Fragment)	4.00E-24	179.33	203.24	34.87	35.29	-2.45	2.20E-10
TRINITY_DN194754_c1_g1_i1	Heat shock factor protein 1	3.00E-61	3530.69	3070.28	1593.95	1805.55	-0.96	2.37E-10

Table S2 Up-regulated genes in low Mg/Ca related to skeletal organic matrix of coral. Listed skeletal organic matrix given in Ramos-Silva et al. (2013).

Gene contig name	blastx (Uniplot-Splot) annotation results	blastx e-value	Expression level 5.2_rep1	5.2_rep2	0.5_rep1	0.5_rep2	Expression ratio (0.5/5.2)
TRINITY_DN186947_c0_g1_i1	Aspartic and glutamic acid-rich protein	7.00E-116	2953.8	2828.7	5020.6	4935.8	1.72
TRINITY_DN186947_c1_g1_i1	Aspartic and glutamic acid-rich protein	2.00E-55	2002.0	1870.7	3370.5	3143.6	1.68
TRINITY_DN198712_c0_g3_i1	Coadhesin (Fragment)	7.00E-07	94.4	123.7	213.9	222.6	2.00
TRINITY_DN202044_c6_g2_i7	Coadhesin (Fragment)	2.00E-13	645.4	707.3	924.2	1193.7	1.57
TRINITY_DN202754_c3_g4_i5	Coadhesin (Fragment)	3.00E-09	264.4	324.2	632.3	554.6	2.02
TRINITY_DN173422_c0_g1_i1	CUB and peptidase domain-containing protein 1 (Fragment)	1.00E-63	494.1	400.0	864.0	1008.3	2.09
TRINITY_DN187257_c0_g1_i1	CUB and peptidase domain-containing protein 1 (Fragment)	2.00E-67	903.9	862.6	1803.3	1818.2	2.05
TRINITY_DN187629_c0_g1_i1	CUB and peptidase domain-containing protein 1 (Fragment)	4.00E-63	710.4	559.8	2252.0	2510.1	3.75
TRINITY_DN188772_c1_g1_i1	CUB and peptidase domain-containing protein 1 (Fragment)	6.00E-44	0.0	0.0	25.3	23.2	N/A
TRINITY_DN190609_c3_g1_i5	CUB and peptidase domain-containing protein 1 (Fragment)	9.00E-63	230.1	247.7	387.2	386.4	1.62
TRINITY_DN196205_c0_g2_i2	CUB and peptidase domain-containing protein 1 (Fragment)	4.00E-10	66.9	77.8	316.4	278.2	4.11
TRINITY_DN196205_c0_g2_i6	CUB and peptidase domain-containing protein 1 (Fragment)	8.00E-15	4.2	7.3	61.1	51.2	9.76
TRINITY_DN196759_c1_g1_i3	CUB and peptidase domain-containing protein 1 (Fragment)	4.00E-52	319.8	400.1	640.1	778.6	1.97
TRINITY_DN199967_c1_g4_i2	CUB and peptidase domain-containing protein 1 (Fragment)	3.00E-35	848.6	752.3	1376.1	1482.3	1.79
TRINITY_DN199967_c1_g5_i3	CUB and peptidase domain-containing protein 1 (Fragment)	5.00E-28	71.5	90.1	289.5	257.3	3.38
TRINITY_DN199973_c1_g3_i1	CUB and peptidase domain-containing protein 1 (Fragment)	7.00E-27	98.9	146.5	299.0	288.6	2.39
TRINITY_DN200686_c1_g3_i4	CUB and peptidase domain-containing protein 1 (Fragment)	3.00E-07	60.8	29.1	128.6	118.0	2.74
TRINITY_DN203718_c1_g1_i1	CUB and peptidase domain-containing protein 1 (Fragment)	3.00E-46	38.2	38.4	109.5	124.5	3.05
TRINITY_DN203718_c1_g1_i2	CUB and peptidase domain-containing protein 1 (Fragment)	1.00E-42	19.3	43.1	148.0	140.1	4.62
TRINITY_DN199967_c1_g5_i1	CUB and peptidase domain-containing protein 2 (Fragment)	1.00E-23	111.5	113.2	309.7	297.1	2.70
TRINITY_DN199967_c1_g5_i2	CUB and peptidase domain-containing protein 2 (Fragment)	3.00E-21	97.2	117.0	282.2	278.7	2.62
TRINITY_DN200686_c1_g3_i3	CUB and peptidase domain-containing protein 2 (Fragment)	6.00E-08	62.0	50.5	167.1	228.3	3.52
TRINITY_DN161539_c0_g1_i1	CUB domain-containing protein	2.00E-11	5024.5	4180.1	6748.2	8618.1	1.67
TRINITY_DN188453_c1_g2_i1	Ectin	7.00E-26	486.8	383.1	1115.9	1474.2	2.98
TRINITY_DN188453_c1_g2_i3	Ectin	2.00E-25	480.3	366.8	897.6	1367.0	2.67
TRINITY_DN189144_c0_g1_i1	EGF and laminin G domain-containing protein (Fragment)	5.00E-10	10.5	5.2	42.9	56.0	6.30
TRINITY_DN199157_c0_g1_i1	Galaxin	6.00E-08	17.3	25.5	1494.3	1685.5	74.43
TRINITY_DN191249_c0_g1_i1	Galaxin 2	3.00E-176	2653.0	2840.1	3522.7	4178.7	1.40

Table S2 Continued

Gene	blastx (Uniplot-Splot) annotation results	blastx	Expression level				Expression ratio
contig name		e-value	5.2_rep1	5.2_rep2	0.5_rep1	0.5_rep2	(0.5/5.2)
TRINITY_DN203804_c1_g1_i2	Hephaestin-like protein	2.00E-93	218.1	291.9	423.3	459.3	1.73
TRINITY_DN187629_c0_g1_i2	MAM and LDL-receptor class A domain-containing protein 1 (Fragment)	2.00E-08	215.4	177.5	478.2	512.0	2.52
TRINITY_DN195790_c0_g1_i2	MAM and LDL-receptor class A domain-containing protein 1 (Fragment)	6.00E-26	156.1	178.3	326.9	413.3	2.21
TRINITY_DN203590_c2_g1_i5	MAM and LDL-receptor class A domain-containing protein 1 (Fragment)	2.00E-16	407.7	473.2	713.6	665.1	1.57
TRINITY_DN187629_c0_g1_i3	MAM and LDL-receptor class A domain-containing protein 2 (Fragment)	7.00E-07	96.9	45.0	245.4	190.8	3.07
TRINITY_DN195790_c0_g1_i1	MAM and LDL-receptor class A domain-containing protein 2 (Fragment)	8.00E-20	289.3	274.7	417.0	550.4	1.72
TRINITY_DN197568_c4_g2_i1	MAM and LDL-receptor class A domain-containing protein 2 (Fragment)	4.00E-08	11451.8	11532.8	16426.5	15350.4	1.38
TRINITY_DN202141_c5_g4_i2	MAM and LDL-receptor class A domain-containing protein 2 (Fragment)	8.00E-08	80.4	53.4	136.6	168.9	2.28
TRINITY_DN202332_c2_g1_i2	MAM and LDL-receptor class A domain-containing protein 2 (Fragment)	2.00E-08	934.5	1036.1	1594.8	1478.2	1.56
TRINITY_DN164412_c1_g1_i1	Mucin-like protein (Fragment)	2.00E-14	161.4	136.1	298.8	293.9	1.99
TRINITY_DN181126_c1_g1_i1	Mucin-like protein (Fragment)	4.00E-95	451.9	587.3	1153.7	1103.8	2.17
TRINITY_DN181126_c1_g1_i2	Mucin-like protein (Fragment)	6.00E-08	19.4	50.8	156.2	78.4	3.34
TRINITY_DN194186_c2_g1_i1	Mucin-like protein (Fragment)	4.00E-11	202.8	145.5	343.1	368.1	2.04
TRINITY_DN195964_c1_g1_i1	Mucin-like protein (Fragment)	1.00E-10	516.4	364.7	882.9	984.2	2.12
TRINITY_DN202377_c1_g1_i1	Mucin-like protein (Fragment)	3.00E-11	129.9	166.4	325.2	295.9	2.10
TRINITY_DN203021_c0_g1_i1	Mucin-like protein (Fragment)	8.00E-14	5225.9	5461.7	9261.9	12415.4	2.03
TRINITY_DN203497_c0_g1_i3	Mucin-like protein (Fragment)	3.00E-169	146.2	144.1	260.3	344.1	2.08
TRINITY_DN204390_c5_g1_i1	Mucin-like protein (Fragment)	4.00E-147	3599.1	3926.3	7196.2	6619.4	1.84
TRINITY_DN204390_c5_g2_i1	Mucin-like protein (Fragment)	1.00E-95	5118.6	6426.7	10339.8	10007.9	1.76
TRINITY_DN140995_c1_g1_i1	Secreted acidic protein 2 (Fragment)	6.00E-30	1620.4	1584.4	3791.8	2935.1	2.10
TRINITY_DN190603_c2_g1_i2	Skeletal aspartic acid-rich protein 1	2.00E-160	1209.4	1317.4	2276.1	2178.9	1.76
TRINITY_DN195315_c3_g1_i1	Skeletal aspartic acid-rich protein 2	6.00E-160	335.0	359.0	1201.3	1071.7	3.28
TRINITY_DN147641_c0_g1_i1	Threonine-rich protein (Fragment)	2.00E-69	2300.4	1999.7	3534.3	3955.8	1.74
TRINITY_DN180406_c0_g1_i1	Uncharacterized skeletal organic matrix protein 2	7.00E-08	199.4	214.5	339.0	361.9	1.69
TRINITY_DN151729_c0_g1_i1	Uncharacterized skeletal organic matrix protein 5	2.00E-27	14.2	34.0	107.3	84.7	3.99
TRINITY_DN190139_c0_g1_i2	Uncharacterized skeletal organic matrix protein 5	4.00E-14	93.2	142.7	217.1	257.2	2.01
TRINITY_DN190857_c1_g1_i1	Uncharacterized skeletal organic matrix protein 5	5.00E-53	1943.8	1648.8	3905.3	4818.9	2.43
TRINITY_DN190857_c1_g1_i2	Uncharacterized skeletal organic matrix protein 5	4.00E-27	97.6	85.7	217.6	251.1	2.56

Table S2 Continued

Gene contig name	blastx (Uniplot-Splot) annotation results	blastx e-value	Expression level 5.2_rep1	5.2_rep2	0.5_rep1	0.5_rep2	Expression ratio (0.5/5.2)
TRINITY_DN199692_c0_g1_i1	Uncharacterized skeletal organic matrix protein 5	3.00E-45	2163.9	2387.7	9071.1	11362.0	4.49
TRINITY_DN201210_c2_g4_i2	Uncharacterized skeletal organic matrix protein 5	1.00E-85	30.7	39.9	126.1	112.0	3.37
TRINITY_DN201210_c2_g6_i1	Uncharacterized skeletal organic matrix protein 5	2.00E-93	39.8	29.5	221.1	320.7	7.82
TRINITY_DN201210_c2_g6_i2	Uncharacterized skeletal organic matrix protein 5	2.00E-77	20.0	30.1	127.7	169.5	5.93
TRINITY_DN204372_c1_g1_i2	Uncharacterized skeletal organic matrix protein 5	2.00E-36	128.3	142.9	306.4	319.0	2.31
TRINITY_DN189073_c1_g1_i1	Uncharacterized skeletal organic matrix protein 8	1.00E-10	435.4	437.4	1046.3	1282.8	2.67
TRINITY_DN189622_c0_g1_i1	Zp domain-containing protein	1.00E-40	1518.5	1510.4	2018.4	2615.1	1.53
TRINITY_DN196376_c3_g4_i1	Zp domain-containing protein	4.00E-38	10074.7	10909.7	16181.8	18877.1	1.67
TRINITY_DN196376_c3_g4_i3	Zp domain-containing protein	1.00E-37	9669.3	10579.4	15785.7	18376.9	1.69
TRINITY_DN200724_c2_g1_i1	Zp domain-containing protein	3.00E-46	772.6	892.1	1183.4	1203.6	1.43
TRINITY_DN200955_c0_g3_i2	Zp domain-containing protein	5.00E-53	1244.6	1292.2	1693.3	1865.8	1.40
TRINITY_DN201880_c1_g1_i1	Zp domain-containing protein	1.00E-34	1645.7	1770.0	2888.3	3230.6	1.79

Table S3 Down-regulated genes in low Mg/Ca related to skeletal organic matrix of coral. Listed skeletal organic matrix given in Ramos-Silva et al. (2013).

Gene	blastx (Uniplot-Splot) annotation results	blastx	Expression level				Expression ratio
contig name		e-value	5.2_rep1	5.2_rep2	0.5_rep1	0.5_rep2	(0.5/5.2)
TRINITY_DN202274_c7_g4_i4	Uncharacterized skeletal organic matrix protein 5	1.00E-11	1680.4	1382.8	398.5	546.5	0.31
TRINITY_DN202274_c7_g5_i1	Uncharacterized skeletal organic matrix protein 5	6.00E-14	1113.3	755.0	214.5	274.5	0.26
TRINITY_DN202274_c7_g4_i3	Uncharacterized skeletal organic matrix protein 5	5.00E-31	1472.8	917.4	241.3	332.9	0.24
TRINITY_DN202274_c7_g5_i2	Uncharacterized skeletal organic matrix protein 5	9.00E-15	1582.5	1212.6	424.3	630.3	0.38
TRINITY_DN188085_c0_g1_i1	Uncharacterized skeletal organic matrix protein 5	2.00E-74	445.3	422.0	96.8	166.5	0.30
TRINITY_DN195053_c1_g1_i1	Zp domain-containing protein	1.00E-47	825.6	755.9	337.2	414.3	0.48
