# Supplementary material for: Differential gene expression in skeletal organic matrix proteins of scleractinian corals associated with mixed aragonite/calcite skeletons under low mMg/Ca conditions
Source: PeerJ. 2019 Jul 15;7:e7241. doi: 10.7717/peerj.7241 (PMC6637933; doi:10.7717/peerj.7241)
Supplement: Supplemental Information 2 — Heat map displaying gene expression levels of detected differentially expressed genes between control and low-Mg/Ca conditions. [file peerj-07-7241-s002.pdf]

Signaling receptor activity (GO:0038023)

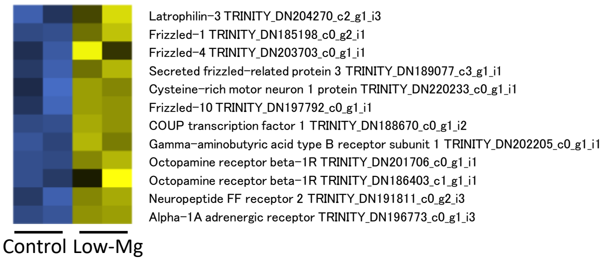

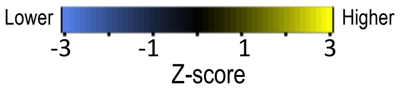

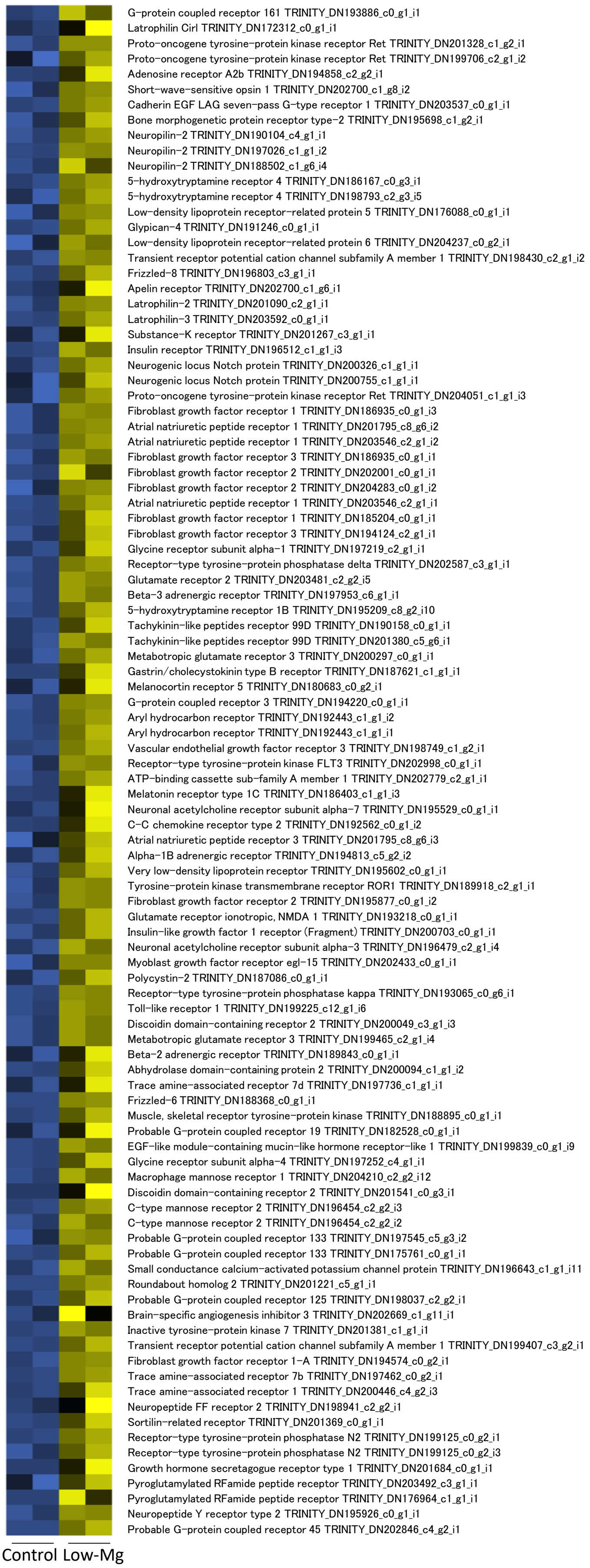

Figure S1. Heat map displaying gene expression levels of detected DEGs between control and low-magnesium conditions. Colors indicate Z-score transformed FPKM values of genes annotated with GO term: signaling receptor activity (GO:0038023); yellow and blue highlights indicate higher and lower expression values, respectively.

Response to endogenous stimulus (GO:0009719)

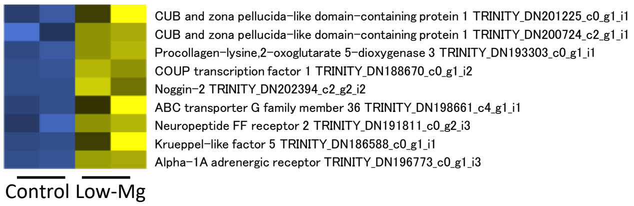

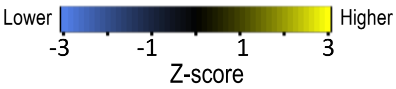

Figure S2. Heat map displaying gene expression levels of detected DEGs between control and low-magnesium condition. Colors indicate Z-score transformed FPKM values of genes annotated with GO term: response to endogenous stimulus (GO:0009719); yellow and blue highlights indicate higher and lower expression values, respectively.

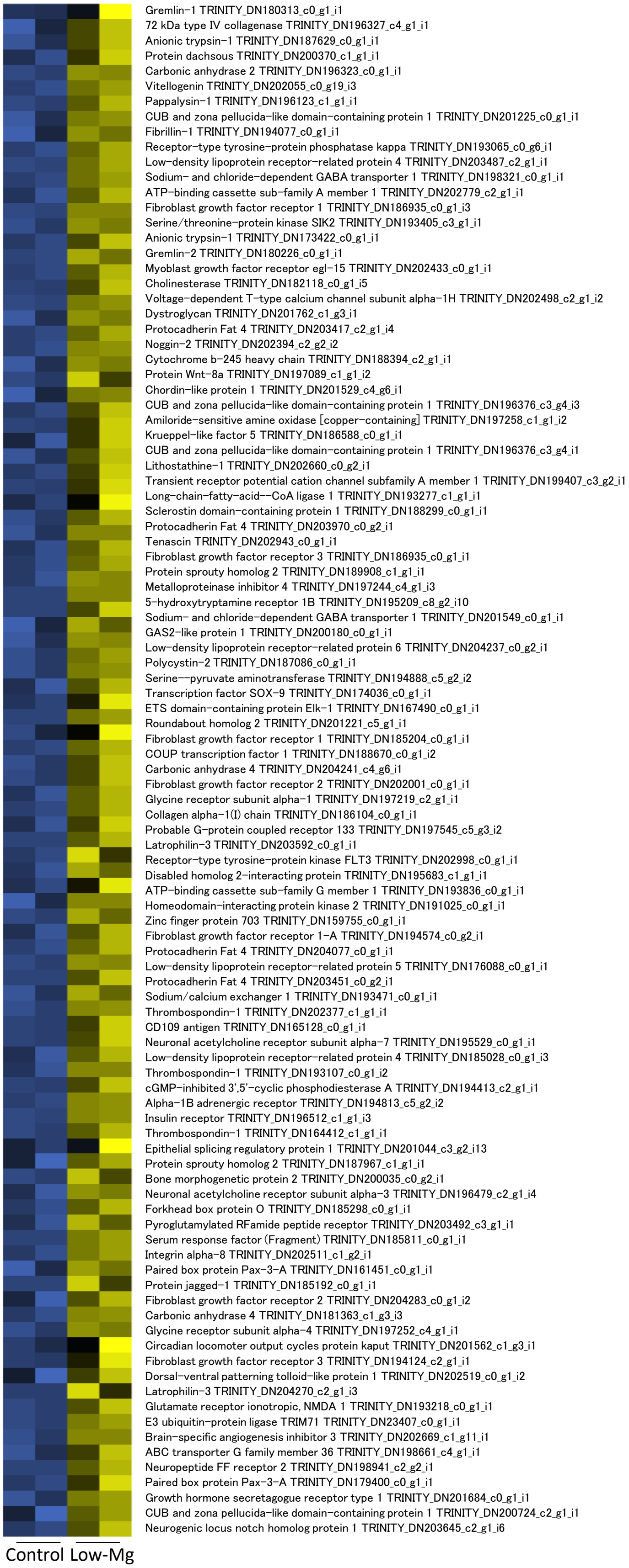

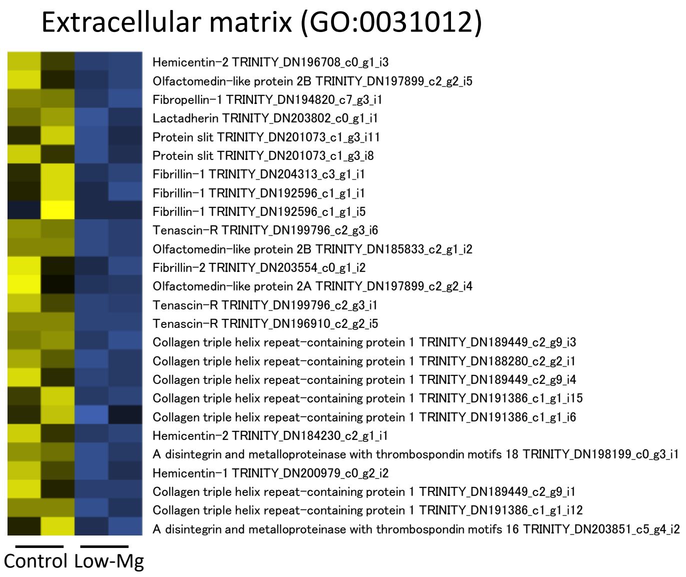

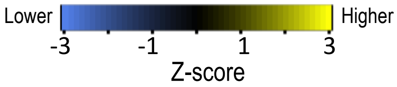

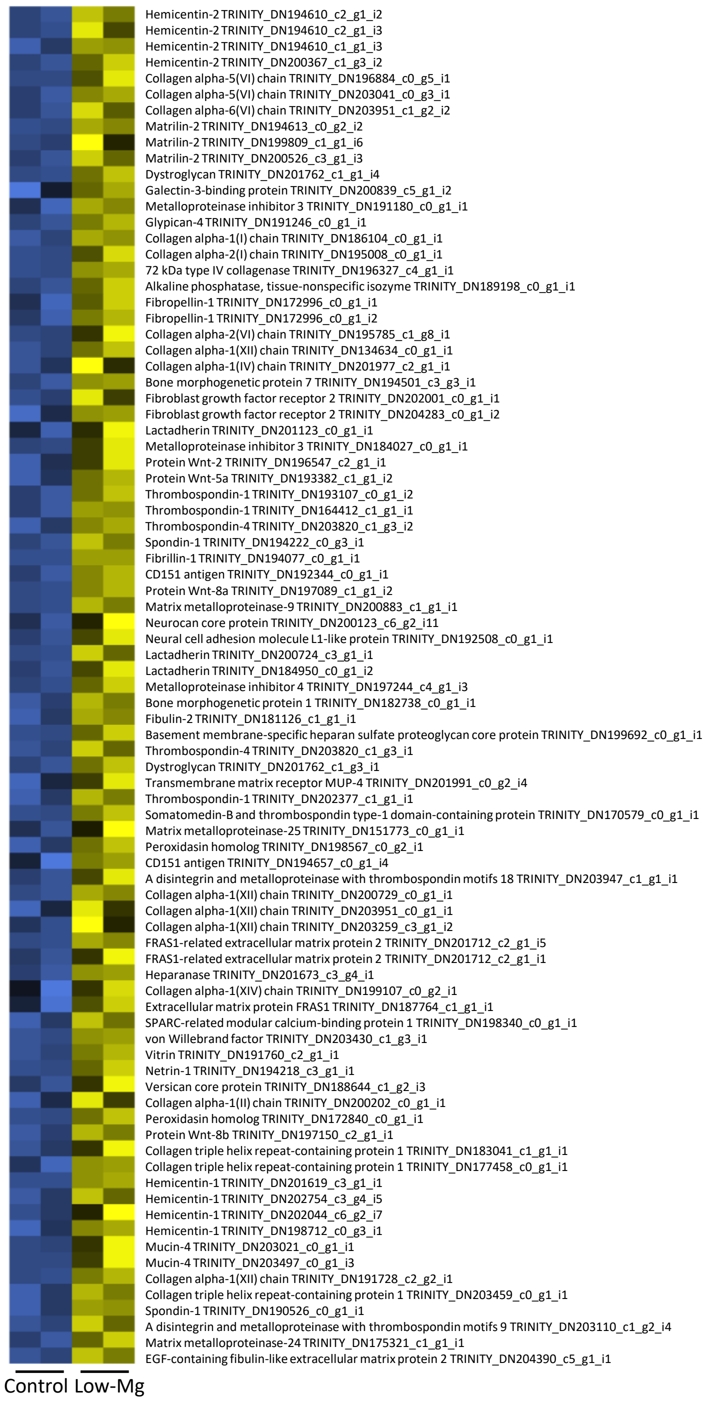

Figure S3. Heat map displaying gene expression levels of detected DEGs between control and low magnesium conditions. Colors indicate Z-score transformed FPKM values of genes annotated with GO term: extracellular matrix (GO:0031012); yellow and blue highlights indicate higher and lower expression values, respectively.

Cell surface receptor signaling pathway (GO:0007166)

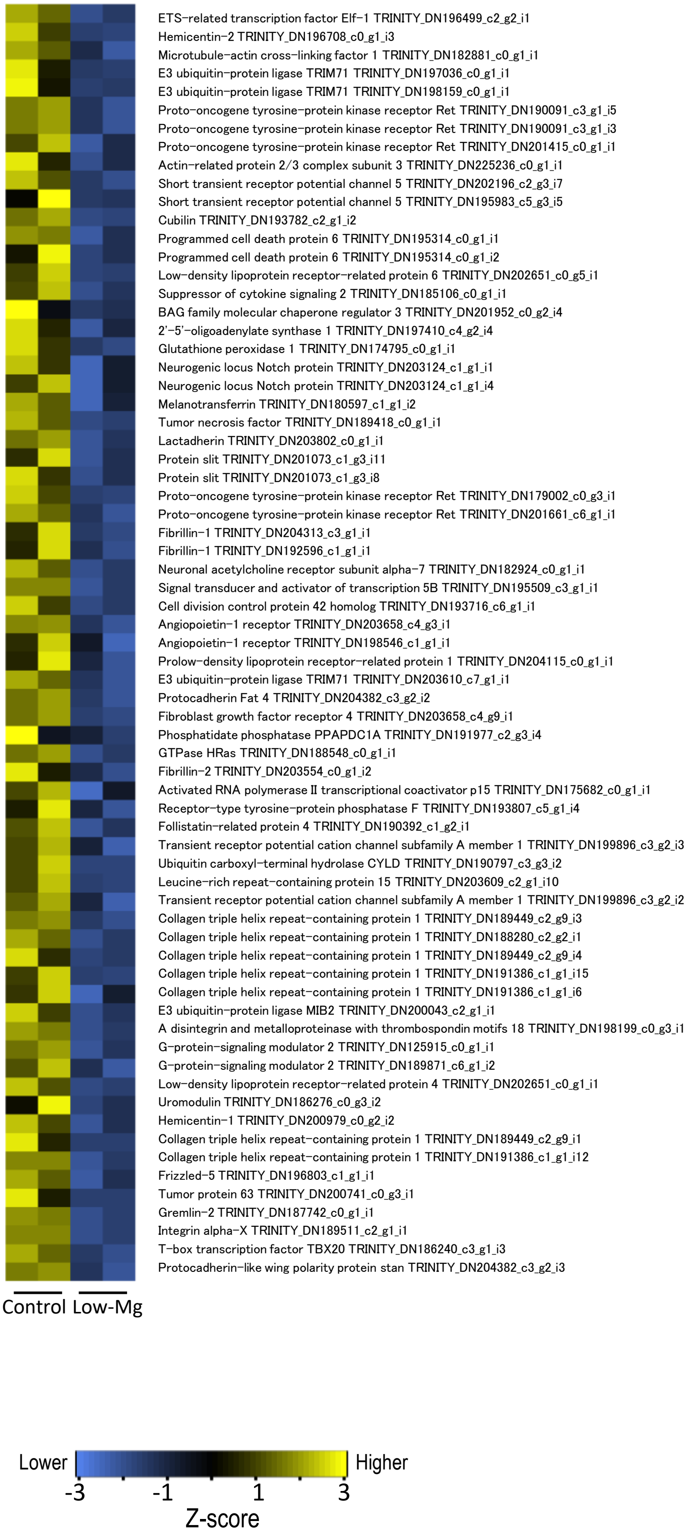

Figure S3 Continued

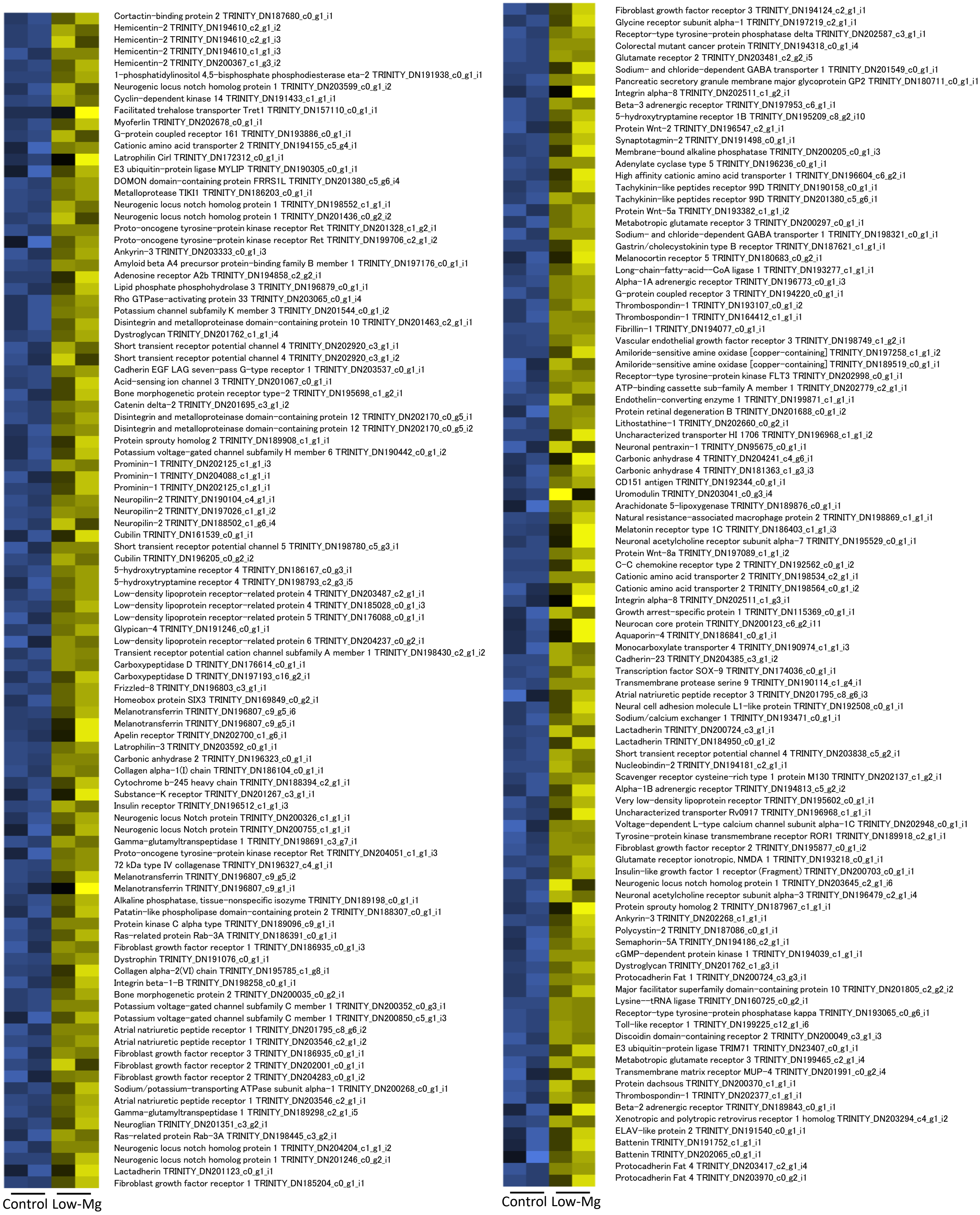

Figure S3 Continued

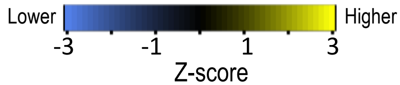

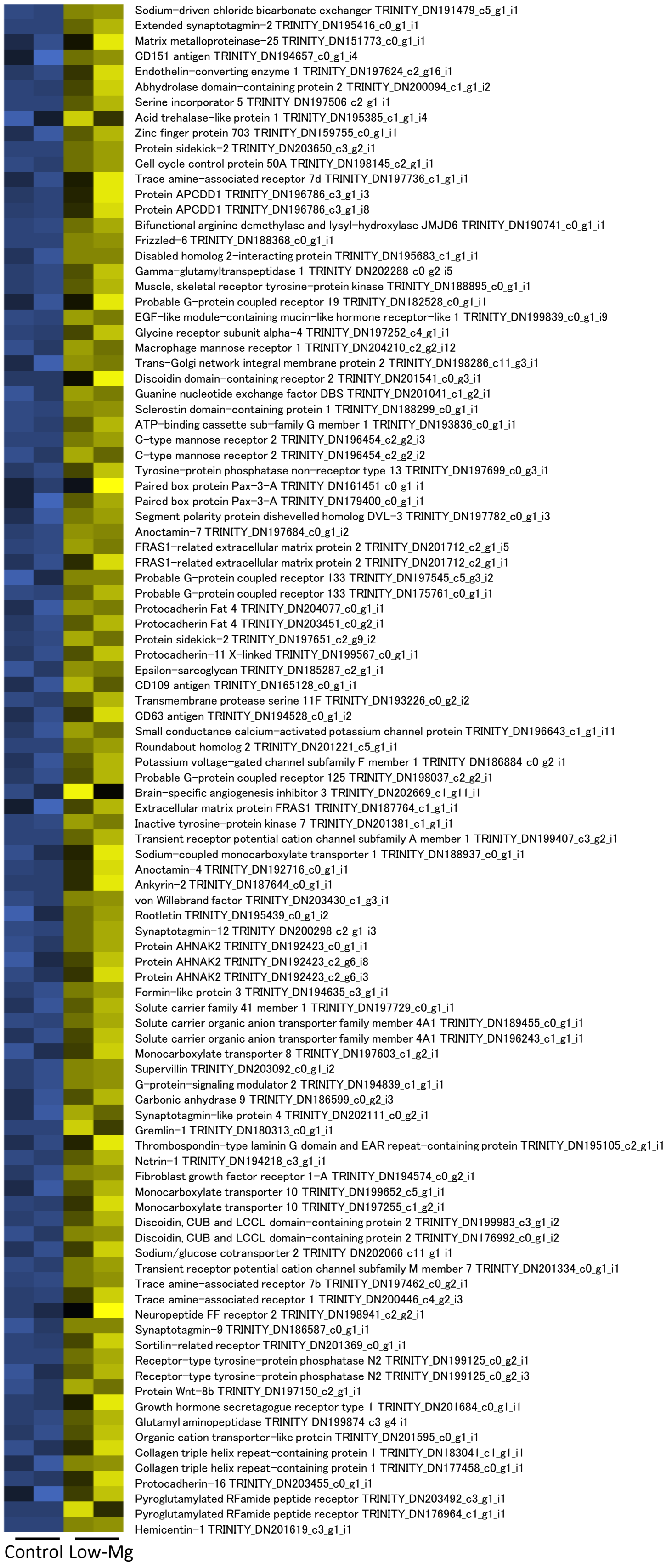

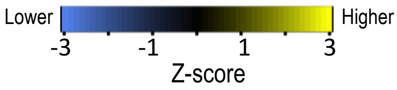

Figure S4. Heat map displaying gene expression levels of detected DEGs between control and low magnesium conditions. Colors indicate Z-score transformed FPKM values of genes annotated with GO term: cell surface receptor signaling pathway (GO:0007166); yellow and blue highlights indicate higher and lower expression values, respectively.

Calcium ion binding (GO:0005509)

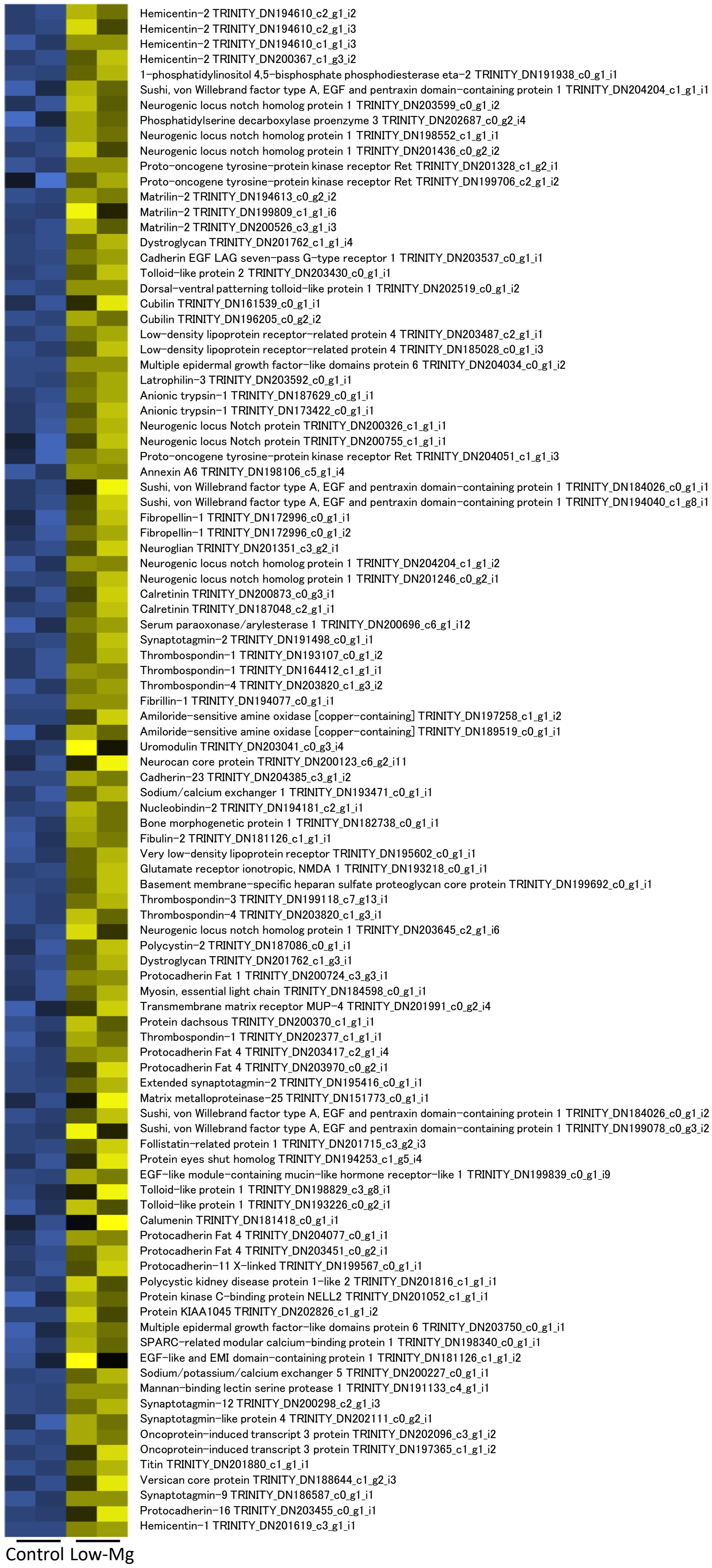

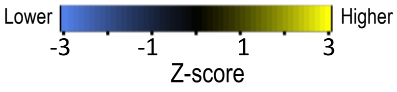

Figure S5. Heat map displaying gene expression levels of detected DEGs between control and low magnesium conditions. Colors indicate Z-score transformed FPKM values of genes annotated with GO term: calcium ion binding (GO:0005509); yellow and blue highlights indicate higher and lower expression values, respectively.

Calcium ion binding (GO:0005509)

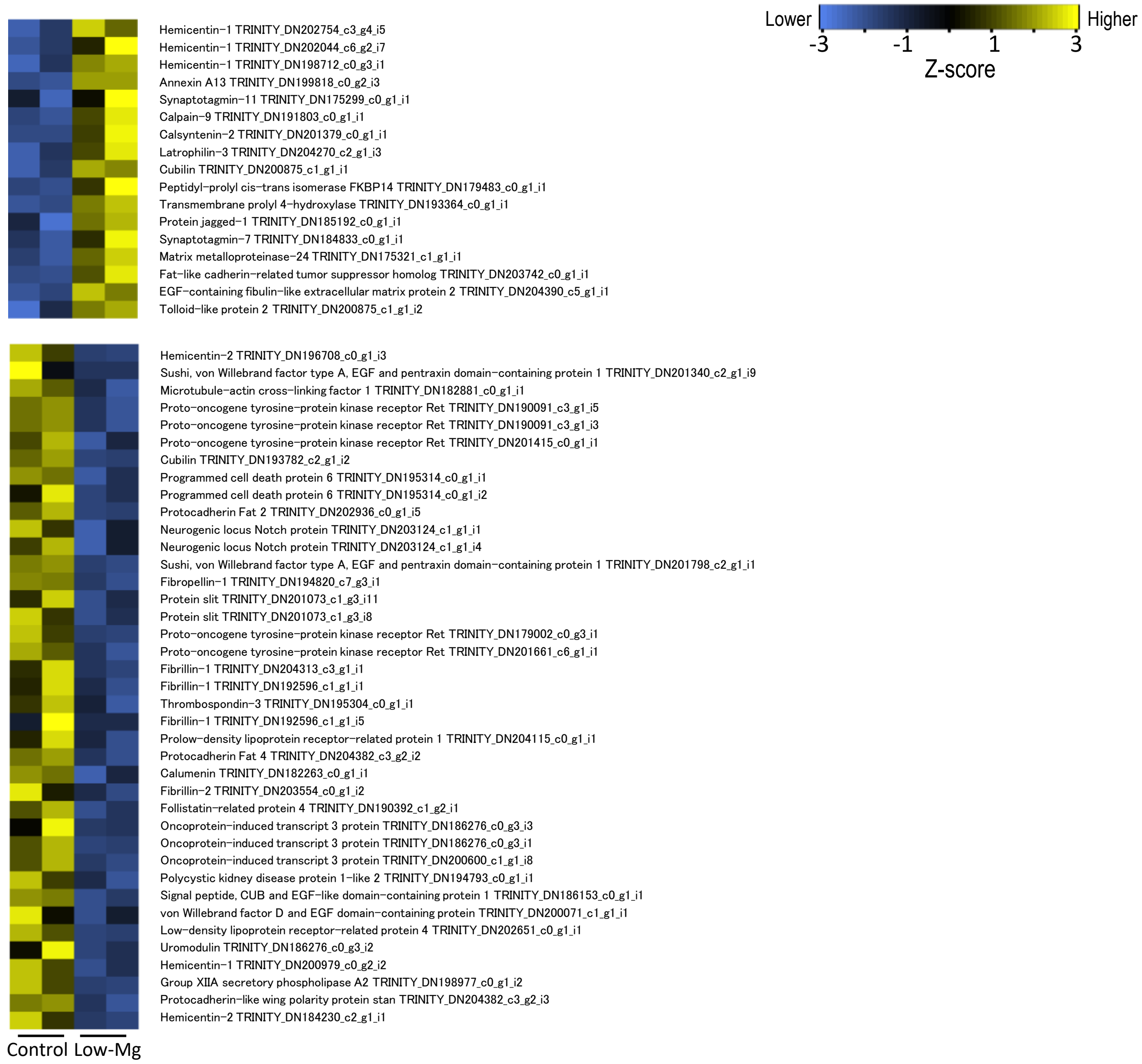

Figure S5 Continued

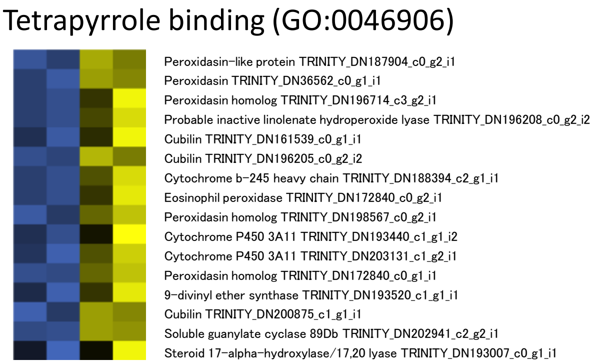

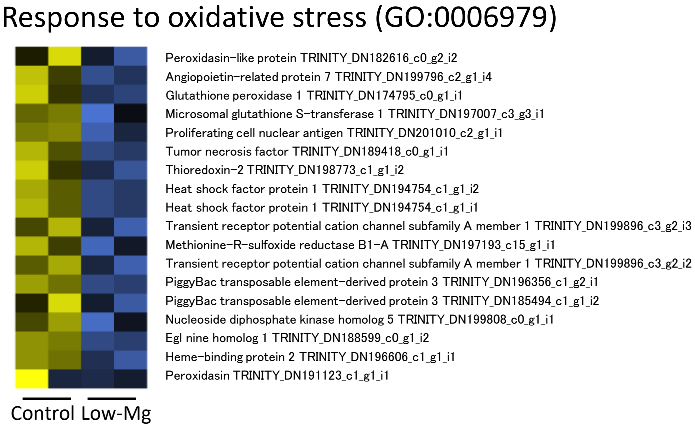

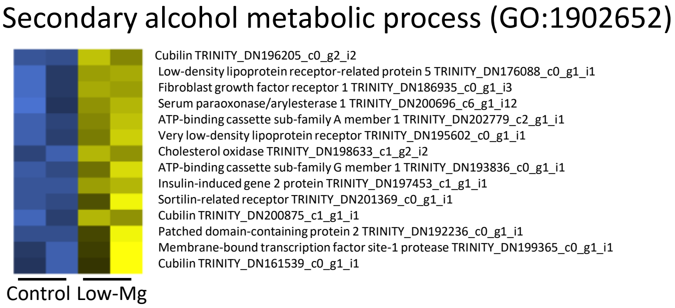

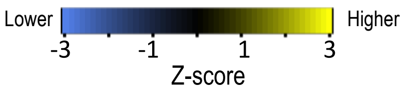

Figure S6. Heat map displaying gene expression levels of detected DEGs between control and low magnesium conditions. Colors indicate Z-score transformed FPKM values of genes annotated with GO terms: tetrapyrrole binding (GO:0046906), regulation of tumor necrosis factor biosynthetic process (GO: 0042534), response to oxidative stress (GO:0006979), sterol metabolic process (GO:0016125), Notch signaling pathway (GO:0007219), and secondary alcohol metabolic process (GO:1902652); yellow and blue highlights indicate higher and lower expression values, respectively.

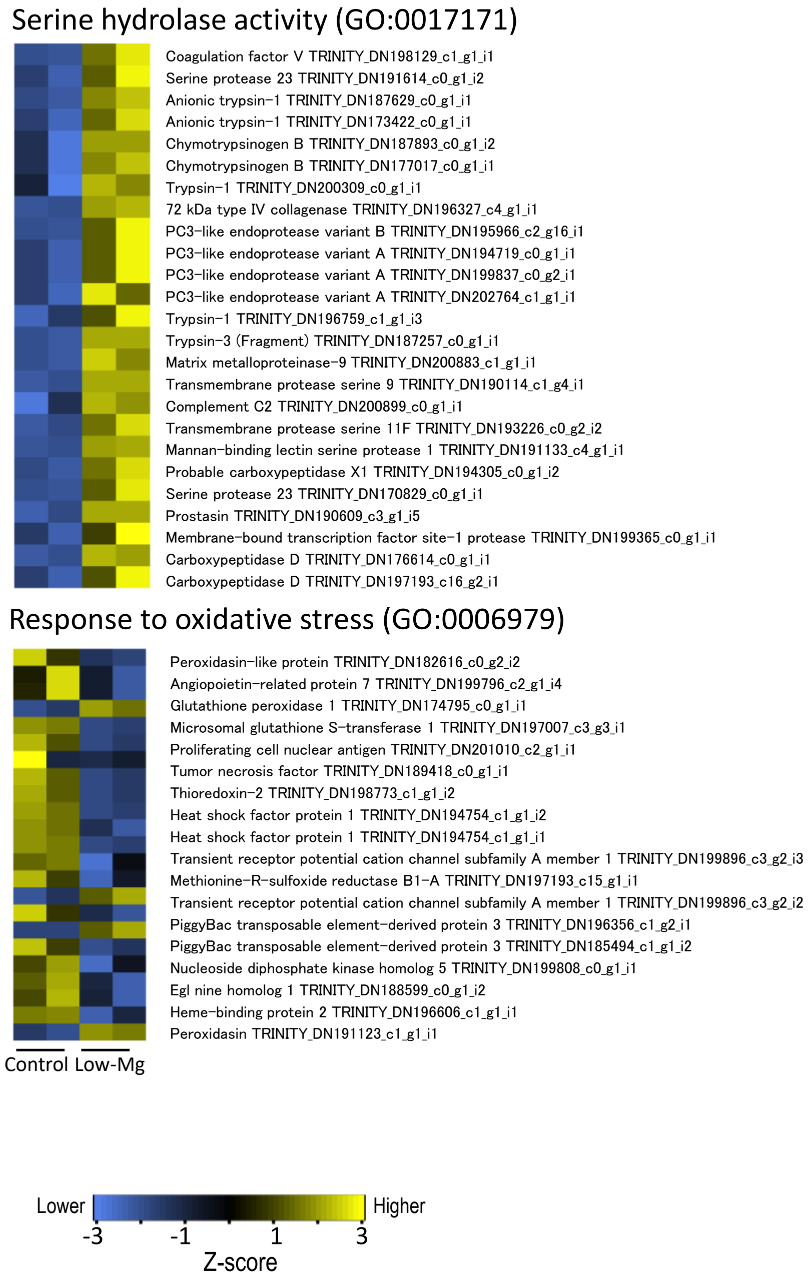

Figure S7. Heat map displaying gene expression levels of detected DEGs between control and low magnesium conditions. Colors indicate Z-score transformed FPKM value of genes annotated with GO terms: serine hydrolase activity (GO:0017171), regulation of tumor necrosis factor biosynthetic process GO:0042534), response to oxidative stress (GO: 0006979), and regulation of MAPK cascade (GO:0043408); yellow and blue highlights indicate higher and lower expression values, respectively.

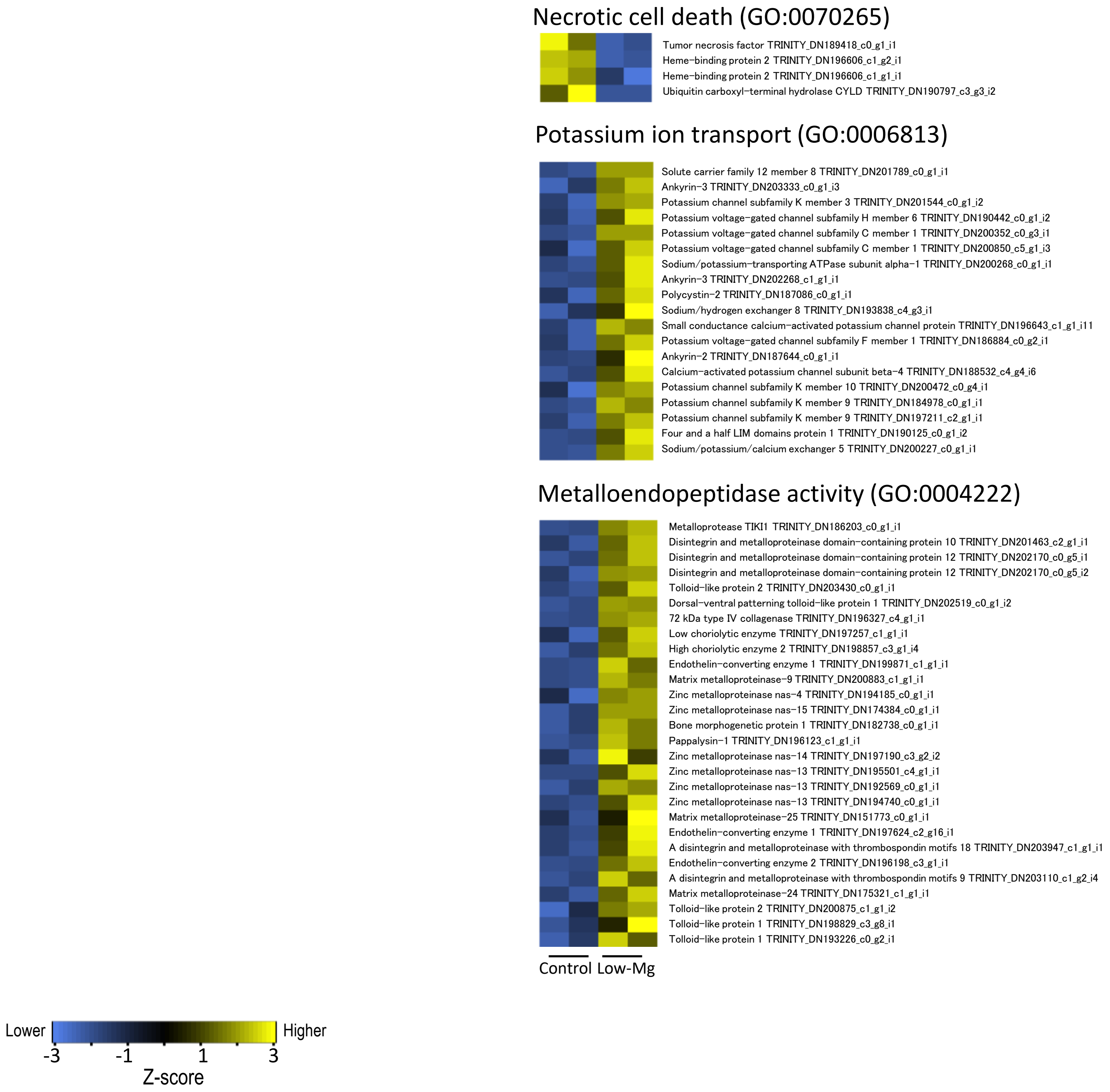

Figure S8. Heat map displaying gene expression levels of detected DEGs between control and low magnesium conditions. Colors indicate Z-score transformed FPKM value of genes annotated with GO terms: positive regulation of nitrogen compound metabolic process (GO:0051173), necrotic cell death (GO:0070265), potassium ion transport (GO:0006813), and metalloendopeptidase activity (GO:0004222); yellow and blue highlights indicate higher and lower expression values, respectively.

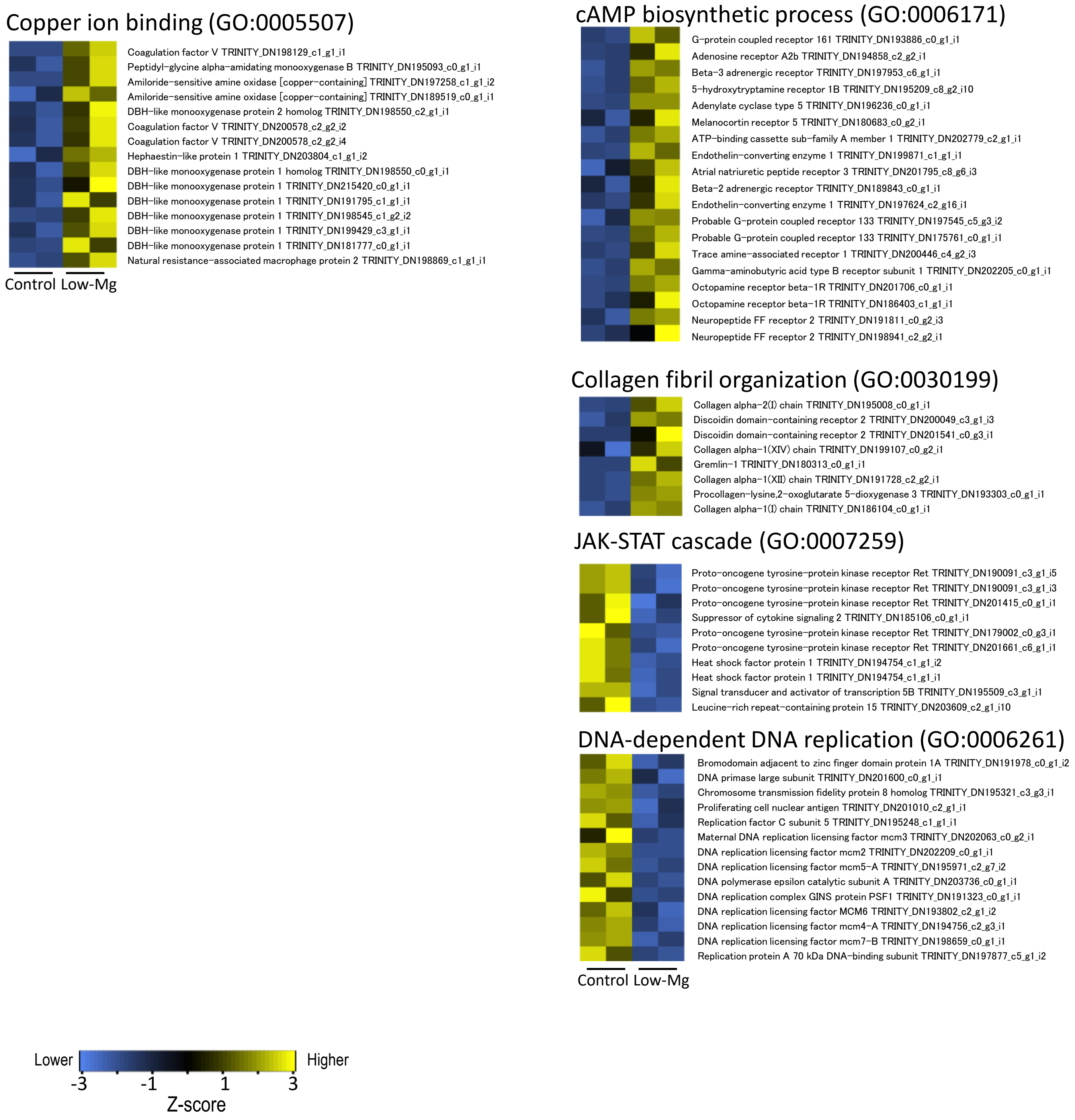

Figure S9, Heat map displaying gene expression levels of detected DEGs between control and low-magnesium condition. Colors indicate Z-score transformed FPKM value of genes annotated with GO terms: macrophage activation (GO:0042116), fibroblast growth factor-activated receptor activity (O:0005007), copper ion binding (GO:0005507), collagen metabolic process (GO:0032963), cAMP biosynthetic process (GO:0006171), collagen fibril organization (GO: 0030199), JAK-STAT cascade (GO:0007259), and DNA-dependent DNA replication (GO:0006261); yellow and blue highlights indicate higher and lower expression values, respectively.

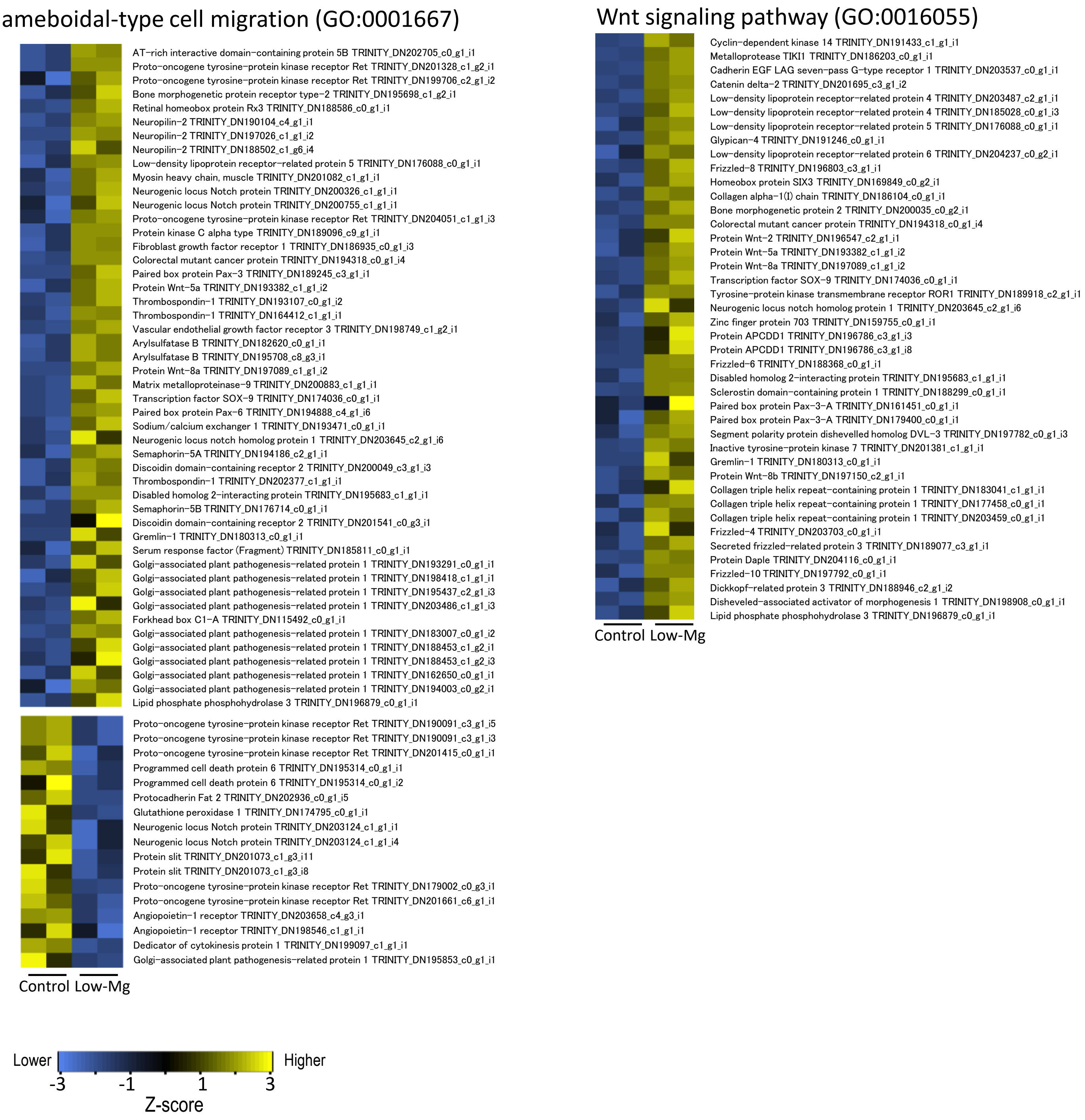

Figure S10. Heat map displaying gene expression levels of detected DEGs between control and low-magnesium conditions. Colors indicate Z-score transformed FPKM value of genes annotated with GO terms: ameboidal type cell migration (GO:0001667) and Wnt signaling pathway (GO: 0016055); yellow and blue highlights indicate higher and lower expression values, respectively.
